# AcuC, a histone deacetylase, contributes to the pathogenicity of *Aeromonas hydrophila*

**DOI:** 10.1002/mbo3.468

**Published:** 2017-03-30

**Authors:** Qingling Jiang, Wenbo Chen, Yingxue Qin, Lixing Huang, Xiaojin Xu, Lingmin Zhao, Qingpi Yan

**Affiliations:** ^1^ Fisheries College Key Laboratory of Healthy Mariculture for the East China Sea Ministry of Agriculture Jimei University Xiamen China; ^2^ State Key Laboratory of Large Yellow Croaker Breeding Ningde China

**Keywords:** *Aeromonas hydrophila*, histone deacetylase AcuC, intracellular survival, pathogenicity, phagocytes

## Abstract

The interactions of pathogens and phagocytes are complex. Our study demonstrated that *Aeromonas hydrophila* B11 can survive in the macrophagocytes of *Tilapia mossambica*. To explore the regulatory processes of *A. hydrophila* survival in the macrophagocytes, we used the mini‐*Tn10* transposon mutagenesis system to build a mutant library by mixing *Escherichia coli* Sm10 (pLOFKm) and *A. hydrophila* B11. In total, 102 mutant colonies were detected, and 11 of them showed reduced survival in macrophagocytes. The mutant with the most severe phenotype, AM73, was chosen for further research. The ORF interrupted by mini‐*Tn10* in AM73 was approximately 960 bp and was deposited in GenBank with the accession number SRP049226. The 319 amino acid protein encoded by the ORF showed a high degree of identity (89%) with proteins in the histone deacetylase/AcuC/AphA family of *A. hydrophila* subsp. *hydrophila* ATCC7966. A strain (AC73) in which the *acuC* mutation was complemented was constructed by generating the recombinant expression plasmid pACYC184‐*acuC* and introducing it into the AM73 mutant strain. Our experiments revealed that strain AM73 was deficient in biofilm formation, adhesion, survival in macrophagocytes, and virulence compared with *A. hydrophila* B11, and all of these biological properties were improved in strain AC73. The expression of 10 significant virulence genes was significantly inhibited in strain AM73. The results indicated that AcuC was an important regulatory protein contributing to the pathogenicity of *A. hydrophila*.

## 1 Introduction

*Aeromonas hydrophila*, an opportunistic pathogen, is widespread in water, domestic animals, and various foods (Çiftci et al., 2016). *A. hydrophila* is reported to be the causative agent of several diseases (da Silva et al., 2012; Peyghan, 2010) in farmed and feral fish as well as gastrointestinal and extraintestinal diseases in humans (Janda & Abbott, 2010). *A. hydrophila* causes high mortality in aquaculture throughout the world and results in extensive economic losses (Reyes‐Becerril, López‐Medina, Ascencio‐Valle, & Esteban, 2011). Therefore, an increasing amount of research is being focused on the virulence of *A. hydrophila*.

The process of infection includes adhesion, colonization, invasion, proliferation in the host, and the secretion of toxins (Huang et al., 2016; Wang et al., 2015). Bacterial adhesion to host surfaces is one of the key steps in the initial infection process (Chen, Yan, Wang, Zhuang, & Wang, 2008; Luo et al., 2016), and many pathogens have been shown to develop the ability to adhere to their hosts (Huang et al., 2015; Lin et al., 2017; Qin, Lin, Chen, Xu, & Yan, 2016). During infection, pathogens are able to neutralize bactericidal and bacteriostatic mechanisms to survive and replicate in various host cells, including macrophagocytes (Beaz‐Hidalgo & Figueras, 2013). The interior of cells can protect pathogens from phagocytes and various antibiotics, which can lead to recrudescent infection (Garzoni & Kelley, 2009; Kaufmann, 2011). Therefore, the ability to survive in host phagocytes is an important virulence factor of pathogens (Qin et al., 2014). Biofilms can not only protect bacteria from host defense mechanisms, including phagocytosis, but also serve as a recalcitrant source of bacteria during antimicrobial therapy (Qin et al., 2014); therefore, biofilm formation is considered to be a virulence factor of pathogens. *A. hydrophila* has conventionally been considered an extracellular pathogen, but previous evidence has demonstrated that it can survive intracellularly (Qin et al., 2014). Other extracellular pathogens, including *Staphylococcus aureus*, have been shown to survive intracellularly (Foster, 2005; Thwaites & Gant, 2011). Currently, research on the genes that are conducive to the intracellular survival of nonobligate intracellular pathogens is limited.

The functions of the three‐gene operon *acuABC* are involved in acetoin catabolism (Grundy, Waters, Takova, & Henkin, 1993). Acetoin utilization A (AcuA) was deduced to be an acetyltransferase of the GNAT family (Sterner & Berger, 2000). The function of acetoin utilization B (AcuB) is unknown, but the acetoin utilization C (AcuC) protein and class I histone deacetylases (HDACs) were shown to be homologs (Thiagalingam et al., 2003). AcuA and AcuC are encoded by the *acuABC* operon, which comprises a protein acetylation/deacetylation posttranslational modification system to control the activity of acetyl‐coenzyme A (Ac‐CoA) synthetase in *Bacillus subtilis* (Leipe & Landsman, 1997). Acetyl‐CoA synthetase, a ubiquitous enzyme, is responsible for the reversible conversion of acetate to Ac‐CoA. The protein acetylation/deacetylation posttranslational modification is an efficient mechanism for controlling the activity of structural proteins, gene expression regulators, and enzymes in response to rapidly changing physiological conditions (Gardner, Grundy, Henkin, & Escalante‐Semerena, 2006). In *Salmonella enterica*, Acs activity is modulated by the protein acetylation/deacetylation system, which is critical to the synthesis of the acetyl‐AMP intermediate from acetate and ATP (Starai, Celic, Cole, Boeke, & Escalante‐Semerena, 2002). Studies of *Escherichia coli* demonstrated that the abundant lysine acetylation may modify or regulate the activities of many enzymes in pivotal metabolic processes and the synthesis of biological building blocks in response to changes in the environment (Gulick, Starai, Horswill, Homick, & Escalante‐Semerena, 2003). The conservation of AcuC suggested the possibility that the *acu*C gene might encode a protein acetylation/deacetylation posttranslational modification system in *A. hydrophila*. However, no studies of the *acu*C gene in *A. hydrophila* have been reported.

In this study, a mini‐*Tn10* transposon mutagenesis system was used to build a mutant library, and a mutant defective in intracellular survival was chosen for further phenotypic analysis. The aim of this study is to explore the possible mechanisms by which *A. hydrophila* survives in macrophages.

## 2 Materials and Methods

### 2.1 Bacteria and culture conditions

The bacteria and plasmids used in this study are listed in Table 1. *E. coli* and *A. hydrophila* were cultured at 37°C and 28°C, respectively, in Luria‐Bertani medium. The bacteria were washed in phosphate‐buffered saline (PBS; pH 7.4) after 24 hr growth. The bacterial density was determined by OD_550_. Antibiotics were added to the medium at the following concentrations: 50 μg/ml ampicillin (Ap); 25 μg/ml chloromycetin (Cm); 100 μg/ml kanamycin (Km); and 50 μg/ml streptomycin (Sm).

**Table 1:** Strains and plasmids

Strains or plasmids	Description	Source
Strains		
* Aeromonas hydrophila*B11	Wild‐type strain (Sm^R^)	(Qin et al., 2014)
* *AM01~AM102	Mini‐*Tn10*Km insertion mutant (Sm^R^Km^R^)	Our study
* *AM73 (*acuC*−)	*AcuC*:: mini‐*Tn10*Km (Sm^R^Km^R^)	Our study
* *AC73 (*acuC*+)	AM73 complemented with pACYC184‐*AcuC* (Sm^R^Km^R^Cm^r^)	Our study
* E. coli*SM10	*thithrleutonAlacYsupErecA*RP4 – 2 ‐ Tc:: Mu:: Km (λ*pir*)	(Qin et al., 2013)
* E. coliDH5*α	F^−^, φ 80d*lacZ*ΔM15, ΔU169 (*lacZYA‐argF*), *deoR, recA1endA1, hsdR17 (rK* ^*−*^ *,mK* ^*+*^ *), phoA, supE44,* λ^*−*^ *, thi ‐1, gyrA96, relA1*	Takara (Qin et al., 2014)
Plasmids		
pMD18 ‐ T	Cloning vector (Ap^R^)	Takara
pLOF/Km	*Tnl0*‐connected transmit plasmid (Km^R^Ap^R^);	(Herrero et al., 1990)
pACYC184	(Cm^R^Tc^R^)	afforded by Prof. Nie
pACYC184 ‐*acuC*	Recombination of pACYC184 and *AcuC* including the promoter and ORF (Cm^R^)	Our study

### 2.2 Preparation of *Tilapia mossambica* macrophage suspensions

Healthy *Tilapia mossambica* individuals were obtained from the market. The macrophages were prepared as previously described (Leung, Low, Lam, & Sin, 1995). In brief, the fish were incubated on ice to reduce activity, and head kidneys were removed, pooled under sterile conditions and then filtered with a 100 μm filter membrane. Leibovitz‐15 medium (Biological Industries, Israel) was used to suspend the samples, and heparin (10 IU/ml), streptomycin/penicillin (100 IU/ml), and 2% fetal calf serum were added. The cell suspension was layered over a 34%/51% discontinuous Percoll gradient. The cell suspension was centrifuged at 400*g* for 30 min at 4°C. The cells above the 34%/51% interface were collected, washed and resuspended in Leibovitz‐15 medium with 10% fetal calf serum, 100 IU/ml streptomycin/penicillin, and 10 IU/ml heparin. After the cell suspension was adjusted to 1 × 10^7^ cells/ml, samples were transferred to six‐well plates (1 ml/well).

### 2.3 Preparation of mucus

Healthy *Anguilla japonica* (297.5 ± 17.6 g) were obtained from the aquaculture farm. Briefly, the skin mucus was prepared by scraping the surface of *A. japonica* (Balebona et al., 1998). The gill mucus was obtained from the gill arches of *A. japonica* by scraping the surface (Lumsden, Ostland, Byrne, & Ferguson, 1993). The intestinal mucus was obtained by scraping the surface of the intestine from *A. japonica* (Yan, Chen, Ma, Zhuang, & Wang, 2007). Mucus was mixed with sterile PBS after incubation at 4°C for 3 or 4 hr. The mixtures were centrifuged for 30 min at 20,000*g* at 4°C. The supernatants were filtered through 0.45 μm and 0.22 μm filters. Then, the protein concentrations of the mucus mixtures were adjusted to 1 mg/ml with sterile PBS using the method of Bradford to measure protein concentration (Bradford, 1976).

### 2.4 Invasion and survival assays of the wild‐type strain in macrophages in vitro

The assays were performed as described previously (Leung, Lim, Lam, & Sin, 1996). In step I, the concentration of bacteria was normalized using colony‐forming units (CFUs) as described by Jin & Pancholi (2006). The exact number of CFUs was determined by plating the bacterial culture on Luria‐Bertani agar plates and counting the resulting colonies. The macrophage suspension was added to six‐well plates and incubated for 2 hr. In step II, the bacteria (100 bacteria/cell) were added, and the mixture was incubated at 28°C for 1 hr. The cells were collected and centrifuged at 100*g* for 5 min at 28°C, and the supernatant was carefully removed. After the macrophages were washed, they were suspended in ice‐cold PBS. The macrophage suspensions received 250 μg/ml gentamycin and were incubated for 20 min at 4°C to remove residual extracellular bacteria, then washed with PBS. The supernatant fluid was withdrawn and plated on solid Luria‐Bertani medium to detect the bacteria in the supernatant. The cells were resuspended in fresh Leibovitz‐15 medium with 10 IU/ml heparin, 10% fetal calf serum and 100 IU/ml streptomycin/penicillin. In step III, the cell suspension was incubated at 28°C in 5% CO_2_ and sampled at 0 hr, 1 hr, 2 hr, 4 hr, 12 hr, and 24 hr. Cells were centrifuged for 5 min at 100*g* and 28°C. After the supernatant was removed, sterile distilled water was used to lyse the cells but not the intracellular bacteria, and the lysate was incubated for 30 min. The cell lysate was diluted 10‐fold with PBS. The dilutions of 10^−3^, 10^−4^, 10^−5^, 10^−6^, 10^−7^ were plated on solid Luria‐Bertani medium and cultivated at 28°C for 24 hr. The resulting bacterial colonies were observed and counted.

### 2.5 Mutagenesis of *A. hydrophila*

*E. coli* Sm10 with pLOFKm (a type of suicide vector) was mated with *A. hydrophila* strain B11 to introduce the mini‐*Tn10*Km transposon into the *A. hydrophila* (Herrero, de Lorenzo, & Timmis, 1990). In brief, *E. coli* Sm10 (mini‐*Tn10*Km) and *A. hydrophila* were mixed at a ratio of 1:4 on 0.22 μm filters and cultivated on tryptone soya agar plates with 3 mmol/L isopropyl β‐D‐1‐thiogalactopyranoside for 4 hr at 28°C. Then, the filters were eluted with trypticase soy broth, and the tryptone soya agar plates were incubated with kanamycin and streptomycin at 28°C for 24 hr. Single colonies were selected for further study of intracellular survival. The procedure was the same as described earlier, except the incubation at the end was 1 hr.

### 2.6 Southern blots

Southern blots were performed as described by Qin et al. (2014). In brief, the genomic DNA of *A. hydrophila* B11 and the mutant strains were extracted with a DNA extraction kit (TaKaRa, Japan). The DNA was digested with SacI (TaKaRa) restriction endonuclease, electrophoresed on a 1% agarose gel, and transferred to a nylon membrane (Liu, Mitsukawa, Oosumi, & Whittier, 1995). Using a digoxigenin‐deoxyuridine‐triphosphate‐tagged probe (Roche, Switzerland), blotting was performed to detect the mini‐*Tn10*Km transposon. A region of the Km^R^ gene was amplified to be the probe. The length of the probe was 176 bp, and the primers used were F_Km3_: 57‐CGG GGA TCG CAG TGG‐37 and F_Km4_: 5'‐TGG GAA GCC CGA TGC‐3' with the DIG‐PCR probe synthesis kit (Roche). Hybridization was performed at 42°C for 16 hr followed by washing and immunological detection with a digoxigenin detection kit (Roche). The results with single bands for the mutants were as expected.

### 2.7 TAIL‐PCR

TAIL‐PCR was performed as previously described by Qin et al. (2014). The random primer was provided by a genomic walking kit (TaKaRa, Japan), and the nested primers were designed on mini‐*Tn10* using the primer Premier Version 5.0 Tool (PREMIER Biosoft International, Palo Alto, CA) (Table 2). The first PCR was performed with the primers L1/R1 and the random primer. The product of the first PCR and the primers L2/R2 were used for the second PCR. The third PCR was performed with the primers L3/R3 and the product of second PCR as template. The thermal cycling conditions are listed in Table 3. The DNA products were purified and cloned into pMD18‐T (TaKaRa, Japan) for sequencing and then analyzed by BLAST, ClustalW and MegAlign (DNAStar) to determine the site interrupted by mini‐*Tn10*.

**Table 2:** Specific primers of TAIL‐PCR

Primer	Sequence (5′→3′)	Application
L1	ATGCTTGATGGTCGGAAGAGGC	upstream sequences
L2	CATCGGGCTTCCCATACAATCG	
L3	ATTATCGCGAGCCCATTTATACCC	
R1	CCTGTTGAACAAGTCTGGAAAGAAATG	downstream sequences
R2	GATCTTGCCATCCTATGGAACTG	
R3	TTACGCTGACTTGACGGGACGG	

**Table 3:** The thermal cycling conditions of TAIL‐PCR

No.	Thermal cycling conditions
1st	
94°C 1 min	1 cycle
98°C 1 min	1 cycle
94°C 30 s; 60°C 1 min; 72°C 150 s	5 cycles
94°C 30 s; 25°C 3 min; 72°C 150 s	1 cycle
94°C 30 s; 60°C 1 min; 72°C 150 s	15 cycles
94°C 30 s; 60°C 1 min; 72°C 150 s	
94°C 30 s; 44°C 1 min; 72°C 150 s	
72°C 10 min	1 cycle
2st	
94°C 30 s; 62°C 1 min; 72°C 150 s	15 cycles
94°C 30 s; 62°C 1 min; 72°C 150 s	
94°C 30 s; 44°C 1 min; 72°C 150 s	
72°C 10 min	1 cycle
3rd	
94°C 30 s; 60°C 1 min; 72°C 150 s	15 cycles
94°C 30 s; 60°C 1 min; 72°C 150 s	
94°C 30 s; 44°C 1 min; 72°C 150 s	
72°C 10 min	1 cycle

### 2.8 Construction of the complemented strain AC73

The *acuC* gene of wild‐type B11 was amplified with the primers *acuC*‐BamHI‐f: *GGA TCC* AGC TGC AAA ACT GGT ACA AG and *acuC*‐2HA‐SalI‐r: *GTC GAC* **TTA CTA GAG GCT AGC ATA ATC AGG AAC TAC GGA TA**G CCG TAG CGT TTG TTC G (the enzyme restriction sites are italicized and the hemagglutinin‐tag is bold). The purified *acuC* gene was digested by BamHI and SalI (TaKaRa) and ligated into pACYC184 to obtain pACYC184‐*acuC*. To construct the complement of the *acuC* mutation, we introduced pACYC184‐*acuC* into the mutant strain AM73. We selected the complemented AC73 strain on plates with chloromycetin and detected the expression of the AcuC protein by western blot (Merino, Rubires, Aguilar, & Tomás, 1996). In brief, the complemented AC73 strain and *A. hydrophila* B11 were heated to 100°C for 5 min. The mixture was centrifuged at 12,000*g* for 15 min at 4°C, and the supernatant was carefully collected to detect the expression of the AcuC protein in cells. The extracellular proteins were obtained as described by Munro, Hastings, Ellis, & Liversidge (1980). Briefly, the cultures of the complemented AC73 strain and *A. hydrophila* B11 grown in Luria‐Bertani medium were spread on sterile cellophane films placed on the surface of solid Luria‐Bertani medium plates. After incubation at 28°C for 48 hr, the bacterial cells were washed off the cellophane sheet with PBS and removed by centrifugation at 12,000 rpm at 4°C for 15 min. Supernatants were sterilized by filtration through a 0.22 μm filter (Sartorius Stedim Biotech, Germany). The intracellular and extracellular proteins were freeze‐dried, dissolved in sterile water, and stained with Coomassie brilliant blue to measure the concentration. The protein solutions were adjusted to the same concentration and heated at 100°C for 5 min. Then, 20 μl samples were separated on a 12% gel and transferred to a polyvinylidenedifluoride membrane. The membrane was blocked with 1% bovine serum albumin and incubated with a 1:1,000 dilution of anti‐hemagglutinin antibody. Membranes were washed and incubated with a 1:3,000 dilution of goat anti‐rabbit horseradish peroxidase‐conjugated secondary antibody, and the signal was detected using a chemiluminescent substrate (PerkinElmer, Waltham). Lysates of *A. hydrophila* B11 were used as controls.

### 2.9 Enzyme activity of the AcuC protein

The AcuC protein is a homologue of the histone deacetylase family of proteins. The activity of AcuC in the intracellular proteins of the wild‐type B11 and the mutant strain AM73 was measured. In this assay, the bacteria were washed twice with ice‐cold PBS and resuspended in sterile distilled water containing protease inhibitors. After being swollen for 60 min, the bacteria were subjected to a freeze‐thaw cycle. The homogenate was clarified by centrifugation at 8,000*g* for 30 min at 4°C; the supernatant was then lyophilized, and the proteins were dissolved in water at a concentration of 1 mg/ml. The Epigenase HDAC Activity Assay Kit (Epigentek, USA) was used to detect the enzymatic activity following the manufacturer's instructions. The proteins were incubated for 90 min with the assay buffer and with the substrate stably coated on the microplate wells. The wells were washed, and the capture antibody was added. Then, the wells were washed again, and the detection antibody and the solution for color development were added. Finally, the enzyme activity was measured colorimetrically by reading the absorbance at 450 nm using a microplate spectrophotometer. The activity of AcuC was proportional to the A_450_.

### 2.10 Bacterial biofilm formation assays of wild‐type strain B11, AM73, and AC73

Biofilm formation was assayed as previously described by Chan & Chua (2005) with some modification. Briefly, 50 μl of an overnight bacterial culture (OD_590_ of 0.2) and 150 μl Luria‐Bertani medium were added to a 96‐well microtiter plate. The plate was incubated at 28°C for 20 hr and washed with PBS and then fixed at 60°C. Two hundred microliters of 1% (wt/vol) crystal violet (Sigma, China) was added, and after 10 min at room temperature, samples were carefully washed with PBS. We added 200 μl of 33% (vol/vol) glacial acetic acid to solubilize the stain and then tested the extent of biofilm formation by reading the absorbance of the solution at 590 nm. The biofilm‐forming ability was calculated using the wild‐type strain as the control.

### 2.11 Bacterial adhesion assays

The assays were carried out as described by Ofek, Courtney, Schifferli, & Beachey (1986) with some modification. Briefly, 100 μl gill mucus, intestinal mucus or skin mucus was added to a microtiter plate and fixed by incubation overnight at 4°C; the unbound mucus was removed by washing the wells twice with 200 μl of sterile physiological saline (0.85% NaCl). Bacterial suspensions were adjusted to OD_550_=0.20 (≈10^8 ^CFU/ml), and equal volumes were added to the wells and incubated at 28°C for 2.5 hr. After washing the wells twice to remove the nonadhering bacteria, the microtiter plate was air‐dried at 60°C for 0.5 hr. Then, 300 μl calf serum was added to each well and incubated at 37°C for 1 hr. After being washed with PBS containing Tween‐20, the microtiter plate was incubated with a 1:100 dilution of anti‐*Aeromonas hydrophila* antiserum (prepared from rabbits in our laboratory) at 37°C for 1 hr. The microtiter plate was washed and incubated at 37°C for 1 hr with 200 μl of a 1:500 dilution of goat anti‐rabbit IgG‐horseradish peroxidase antibody. After the wells were washed, 100 μl fresh o‐phenylenediamine‐H_2_O_2_ was added to the wells, and the samples were allowed to react at room temperature for 0.5 hr in the dark. The reaction was terminated by adding 50 μl 2 mol/l H_2_SO_4_ to each well. The OD_492_ was measured using a microtiter plate reader (Thermo Scientific Varioskan Flash).

### 2.12 Bacterial survival assays of AM73, AC73, and the wild‐type strain

The assays were performed according to the procedure described for the invasion and survival assays in macrophages described above except that the cell suspensions were incubated at 28°C in 5% CO_2_ only for 0 hr and 1 hr. The results at 0 hr and 1 hr indicate the ability of the bacteria to invade and survive, respectively, in macrophages.

### 2.13 Microscopic analyses

To construct the fluorescence labeled wild‐type B11, the EX‐EGFP‐B01 (pReceiver‐B01 with enhanced green fluorescent protein) was introduced into the bacteria and measured by direct visualization with an inverted microscope using UV for excitation (Leica DM‐4000B). The invasion and survival assays were performed as described above. The cell suspension was incubated at 28°C in 5% CO_2_ for 0 hr and 1 hr and observed with a confocal microscope to evaluate the status of *A. hydrophila* B11 in cells.

### 2.14 Bacterial infection assays

Infection of *Danio rerio* was performed as described by (Neely, Pfeifer, & Caparon (2002). The infection was carried out using wild‐type B11, AM73, and AC73, and PBS was used as a control. Briefly, the bacterial concentration was adjusted to OD_550_=0.20 (10^8^ CFU/ml). The *Danio rerio* were incubated on ice to reduce their activity. Twenty fishes per group were injected intraperitoneally using an ultrafine insulin syringe to inject 30 μl of PBS or suspensions of B11, AM73, or AC73 into each fish. The head kidneys of infected fishes were homogenized in PBS and spread on Luria‐Bertani agar plates. The identity of individual colonies was verified by 16S rRNA gene sequence analysis. The log‐rank test was used to evaluate mortality.

### 2.15 Quantitative real‐time PCR

To test the effect of the *acuC* mutation on other significant virulence genes, quantitative real‐time reverse transcription‐PCR was performed to quantify the expression of the outer membrane protein genes (*ompA* and ompTS), the type Ш secretion system gene (*ascV*), pilin and flagellar family protein genes (*traA*,* flgE*,* flgL*, and *pilB*), the serine peptidase gene (*degQ*), the type VI secretion system‐related protein gene (*vash*), the lipopolysaccharide gene (*rfaF*), the adhesin gene (*cblA*), and the aerolysin and hemolysin genes (*hlyA* and *aerA*) in *A. hydrophila* B11 and the mutant, AM73, as described by Lü et al. (2015) with some modifications. Total RNA was isolated from *A. hydrophila* B11 and AM73 with Trizol. The first‐strand cDNA was synthesized from the total RNA using the PrimeScript^®^ RT kit. The *acuC* gene in the wild‐type strain was detected by PCR with the cDNA as the template. The sequences of 13 virulence genes were obtained from the NCBI. The primers designed using Premier 5.0 software are listed in Table 4. The analysis was performed on the Step One Plus Real‐Time PCR system (ABI, USA) using SYBR green I fluorescent dye. The reactions were performed in a 10 μl volume containing 0.2 μl SYBR Green I, 5 pmol/l primers and approximately 50 ng cDNA. The cycling parameters were 95°C for 10 min, followed by 45 cycles of 95°C for 20 s, 55°C for 20 s, and 72°C for 20 s. Threshold cycles and dissociation curves were determined with Rotor Gene 6,000 software, and the gene expression levels were normalized to those of 16S rRNA (Kong et al., 2015).

**Table 4:** Primers designed for qRT‐PCR

Gene	Primer	sequence (5′ → 3′)
*acuC*	Ac‐F	AGGACGATGCCTACCTCACC
	Ac‐R	GCGTTTGTTCGCCTCTTCA
*cblA*	Ad‐F	CCGAGGCGTTCTATGTGCA
	Ad‐R	TTGGTCAGGTAGCCGGTGAT
*aerA*	Ae‐F	GGTCTGTGGCGACAAGTATCG
	Ae‐R	AGAGCAGACAGAGTCGGTATTTCTC
*ascV*	As‐F	GGGTATTCACCTGCGTTTCA
	As‐R	GATGTTCATTAGCGACCCACA
*hlyA*	Hl‐F	CCGCCCAGTCCTTCATCTAT
	Hl‐R	AGGGTCCGTAGGCTCACATT
*ompA*	Om‐F	CTCACGATCTGGGTGACTTTG
	Om‐R	CGCCGTTGATGGACTTGA
*ompTS*	Pr‐F	AATGGCTCCTTCCCTGATCG
	Pr‐R	TGGCACCCTGGTTCTCGTAA
*vash*	Va‐F	AAACTGGCACGGGGAAAGAG
	Va‐R	GCTTGTAAGGTGAGCGGCATAT
*traA*	Tr‐F	GTGATGGTCGTCGCCTTTCT
	Tr‐R	GATAACCTTCTCCGCATTTTCC
*pilB*	Pi‐F	CTAATGCGAATGCAGCACGTA
	Pi‐R	CGCTTCAACAGTTCCAACCA
*rfaf*	Rf‐F	TACCTGGCACTGGCCTATCC
	Rf‐R	CTCGTCGAGGTGCTTTTGTG
*degQ*	De‐F	CACCGAGCTTACCTCCGAAAT
	De‐R	CCGCCTTCTTCAGCGTGAC
*flgE*	Fe‐F	CCCGCTCAGACATTGGAGAT
	Fe‐R	GTCGCATTGCTGTAGGTCGC
*flgL*	Fl‐F	GCCCCAGAACAACAACATCC
	Fl‐R	GCCGCATCCTCTTTTGACA
16s	16s‐F	GGGGAGTACGGTCGCAAGAT
	16s‐R	CGCTGGCAAACAAGGATAAGG

### 2.16 Statistical analysis

The results were statistically analyzed by ANOVA using SPSS18.0 and are reported as the means ± *SD*. A value of *p *<* *.05 indicated a significant difference between samples.

## 3 Results

### 3.1 Invasion and survival assays of the wild‐type bacteria in macrophages in vitro

**Figure 1:**
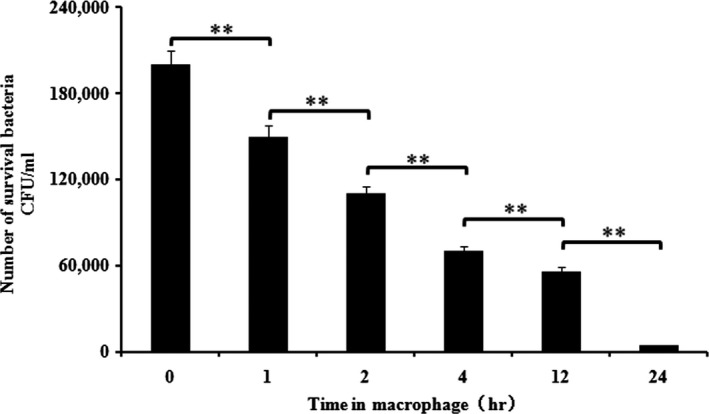
The survival of *A. hydrophila* B11 in macrophages. The *X*‐axis represents the time in the macrophages, and the *Y*‐axis represents the number of surviving bacteria per ml of cells. The number of bacteria that survived in the macrophages gradually decreased with time. However, 2.1% of the invasive bacteria still survived after 24 hr. The data are expressed as the mean ± standard deviation and were statistically analyzed using SPSS18.0. The differences between the mean values were determined by an analysis of variance (ANOVA). Values denoted by different numbers of asterisks were significantly different when compared by ANOVA (“**” *p *<* *.01; “*” *p *<* *.05)

To validate the idea that *A. hydrophila* could invade and survive in macrophages, we analyzed the invasion and survival of *A. hydrophila* B11 in macrophages. The data indicated that 2.0 × 10^5^ CFU/ml *A. hydrophila* B11 invaded the cells, and 1.5 × 10^5 ^CFU/ml survived in the macrophages after 1 hr. The survival at 12 hr and 24 hr was 28% and 2.1%, respectively, of the invading bacteria (Figure 1). Thus, we determined that *A. hydrophila* B11 had the ability to invade macrophages, and some bacteria survived more than 24 hr.

### 3.2 The isolation of mutant strains

**Figure 2:**
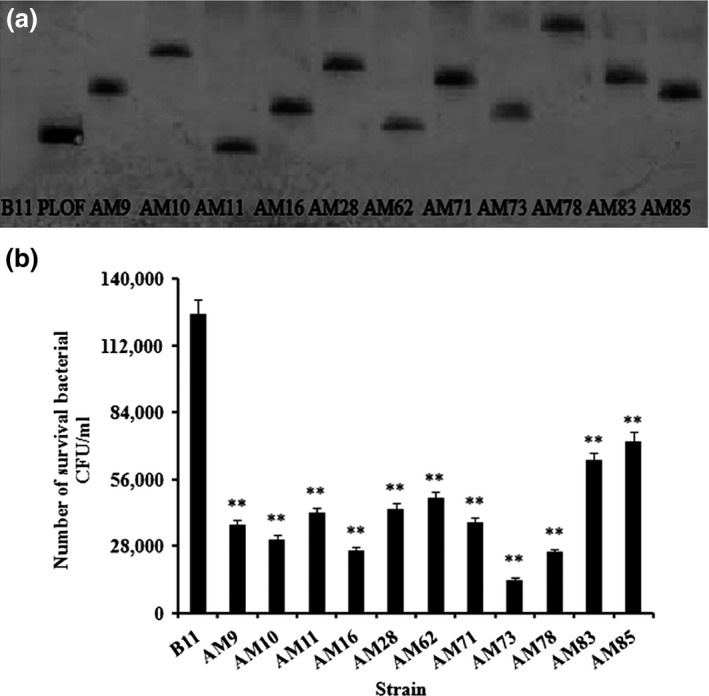
(a) Southern blotting of DNA from wild‐type B11 and mutant strains after digestion with restriction enzymes. The DNA extracted from wild‐type B11 and the mutant strains was digested with PstI. The restricted DNAs were electrophoresed, transferred to nitrocellulose, and hybridized to mixed DIG‐High Prime. The single band indicated that only a single mini‐Tn10 transposon was inserted in the mutants. (b) The number of surviving mutant bacteria in 1 ml of cells was analyzed 1 hr after invasion of the macrophages. Wild‐type B11 was the control. Values denoted by different numbers of asterisks were significantly different when compared by ANOVA (“**” *p *<* *.01; “*” *p *<* *.05)

To screen for crucial virulence genes of *A. hydrophila*, a mutagenesis library was constructed by introducing the mini‐*Tn10*Km transposon on the suicide plasmid pLOFKm into *A. hydrophila* B11. The mutants were selected on tryptone soya agar containing 100 μg/ml kanamycin and 50 μg/ml streptomycin. Eleven colonies that exhibited significant declines in intracellular survival were selected from 102 mutants. The survival rates of the 11 mutant strains were lower than that of the wild‐type B11 (*p *<* *.01) (Figure 2a). The survival rates of AM78, AM73, AM71, AM16, AM10, and AM9 were lower than those of the other mutants. These mutants have been shown to carry mutations of *flgE*,* acu*C, *asmA*,* glpC*,* ahHr*, and *merR*, respectively. The AM73 mutant, which had the lowest survival among the mutants, was chosen for further examination, and the remaining mutants were studied in our other research.

### 3.3 Southern blot analysis

Southern blots were performed to detect the number of mini‐*Tn10*Km transposons inserted into the bacterial genome. The mutants and the positive control pLOFKm showed only one copy of the transposon, whereas the wild‐type strain B11 showed no transposon signal (Figure 2b). Thus, we determined that the insertion of a single copy of the transposon mini‐*Tn10*Km could lead to a mutation.

### 3.4 Identification of the *acuC* gene

**Figure 3:**
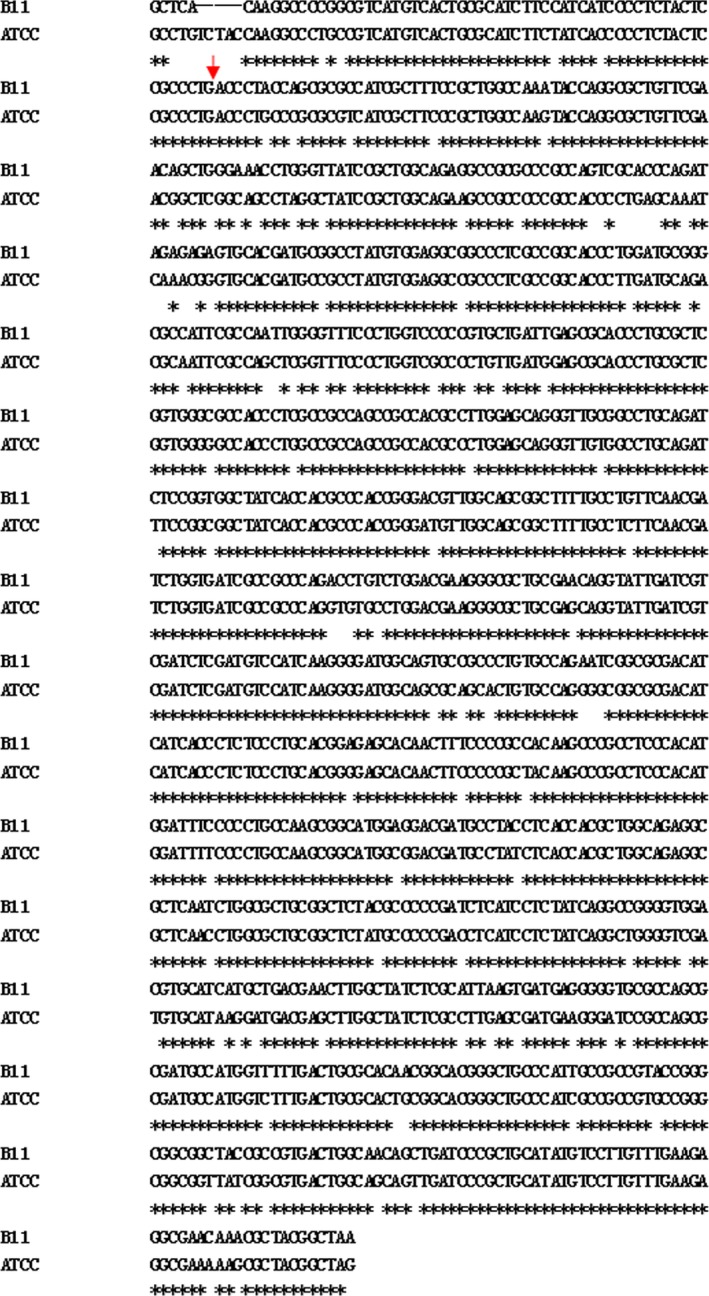
The nucleotide sequence alignment of the ORFs of *A. hydrophila* B11 to *Aeromonas hydrophila* subsp. *hydrophila* ATCC 7966. The initiation codon is shown in bold, and the insertion site is indicated in red arrow

To identify the gene that was mutated in AM73, a series of experiments were performed. The sequence 1,500 bp upstream of the inserted transposon and the sequence 2,000 bp downstream of the inserted transposon were analyzed. The results showed that the *acuC* gene, including the 960 bp ORF, was interrupted by mini‐*Tn10* (GenBank accession numbers: SRP049226) (Figure 3). The protein encoded by the ORF was deduced to be 319 amino acids, and it showed the highest identity (89%) with the histone deacetylase/AcuC/AphA family proteins of *A. hydrophila* subsp. *hydrophila*, ATCC 7966.

### 3.5 Complementation

**Figure 4:**
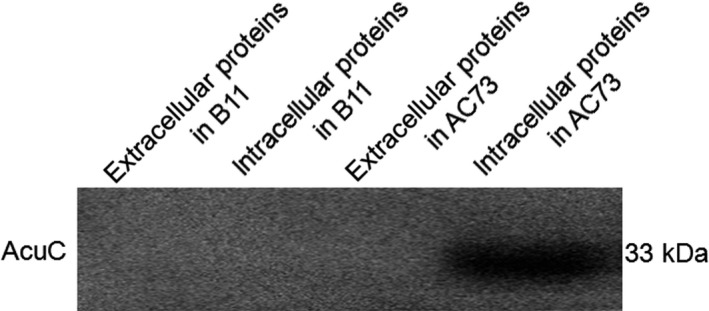
Western blot analysis showing a band of approximately 33 kb exclusively in the intracellular protein of AC73. The intracellular and extracellular protein of the wild‐type B11 was used as the control. Each lane was loaded with 20 μg protein

To validate the effects caused by the mutation in AM73, *acuC* with a hemagglutinin‐tag and pACYC184 were modified to generate the pACYC184‐*acuC* recombinant expression plasmid, which was introduced into the mutant strain AM73 to restore the expression and function of *acuC*. We assessed the AcuC protein in the extracellular and intracellular proteins of AC73 by western blot with the intracellular proteins of B11 as the control. The size of AcuC is approximately 33.02 kDa. The corresponding band was absent in the extracellular proteins of B11, but it was present in the intracellular proteins of AC73. The band probed with anti‐hemagglutinin antibody only appeared in the intracellular proteins of AC73 because the hemagglutinin‐tag is located on the pACYC184‐*acuC* recombinant plasmid (Figure 4). These results confirmed the integrity of *acuC* in AC73 and the AcuC protein as an intracellular protein in *A. hydrophila*.

### 3.6 Enzyme activity

**Figure 5:**
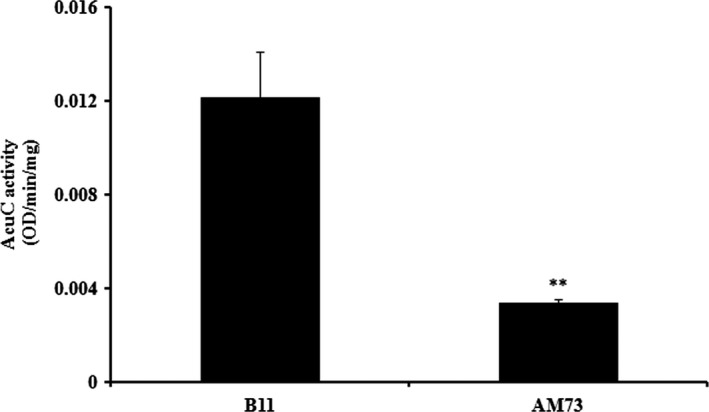
The HDAC enzyme activity in the intracellular proteins of the wild‐type B11 and the mutant AM73 was measured. The protein concentration was 1 mg/ml. Values denoted by different numbers of asterisks were significantly different when compared by ANOVA (“**” *p *<* *.01; “*” *p *<* *.05)

To assess the function of *acuC* in *A. hydrophila*, the corresponding enzyme activity was measured. The activity of the intracellular protein in the B11 strain was threefold higher than the enzyme activity in AM73 (Figure 5). This result demonstrated that the AcuC protein had HDAC enzyme activity.

### 3.7 Biofilm formation

**Figure 6:**
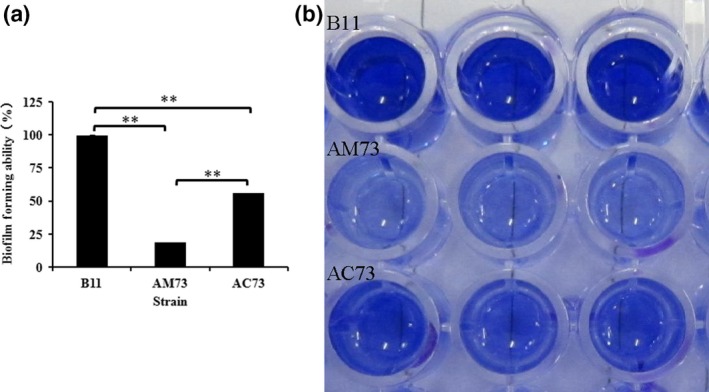
(a) The biofilm‐forming ability of wild‐type B11, AM73, and AC73. Values denoted by different numbers of asterisks were significantly different when compared by ANOVA (“**” *p *<* *.01; “*” *p *<* *.05). (b) The picture shows the color of crystal violet dissolved in 33% acetic acid

To ascertain whether *acuC* could affect other virulence factors as well as survival, biofilm formation by wild‐type B11, AM73, and AC73 was quantified by absorbance readings (A_590_). We found that the biofilm formation by AM73 was only 18.75% of that of the wild‐type B11 and that the biofilm formation of the complemented strain was 56.25% of that of the wild type (Figure 6a). The extent of biofilm formation can be determined based on the depth of color generated by adding glacial acetic acid to the slides. The color of the B11 strain and the complemented strain AC73 was darker than that of AM73 (Figure 6b). This result demonstrated that *acuC* affects the biofilm formation of *A. hydrophila* and that *acuC* might be involved in the regulation of multiple virulence factors.

### 3.8 Bacterial adhesion

**Figure 7:**
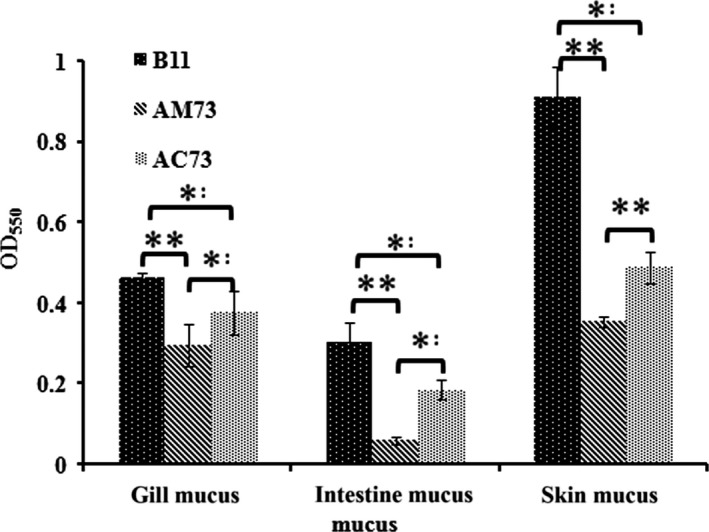
The adhesion of B11, AM73, and AC73 to gill mucus, intestinal mucus, and skin mucus. Values denoted by different numbers of asterisks were significantly different when compared by ANOVA (“**” *p *<* *.01; “*” *p *<* *.05)

To investigate the ability of *acuC* to affect other virulence factors in addition to survival, the adhesive capacity of wild‐type B11, AM73, and AC73 was also tested and quantified. We employed gill mucus, intestinal mucus, and skin mucus for this test. The adhesion of wild‐type B11, AM73, and AC73 was 0.85, 0.43 and 0.64, respectively, in the gill mucus, 0.68, 0.28, and 0.49 in the intestinal mucus, and 1.61, 0.64, and 1.15 in the skin mucus. The results showed that the largest number of *A. hydrophila* adhered to the skin mucus and that the adhesive capacity of the AM73 mutant was significantly less than that of the wild type (Figure 7). These results indicated that the *acuC* gene plays an important role in bacterial adhesion and that *acuC* might be involved in the regulation of multiple virulence factors.

### 3.9 Invasion and survival assays of AM73, AC73, and the wild‐type strain

**Figure 8:**
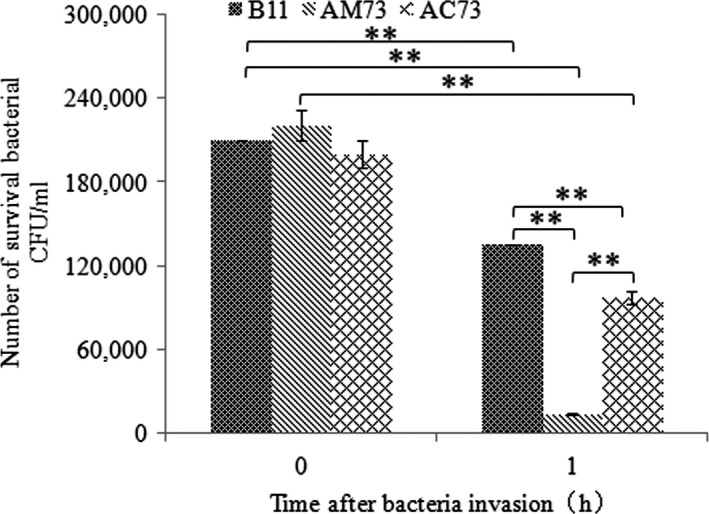
The invasion and survival of B11, AM73, and AC73 in macrophages. The *X*‐axis represents time after bacterial invasion; the *Y*‐axis represents the number of surviving bacteria per ml of macrophages. Values denoted by different numbers of asterisks were significantly different when compared by ANOVA (“**” *p *<* *.01; “*” *p *<* *.05)

The ability of B11, AM73, and AC73 to invade and survive in macrophages was compared to assess the role of *acuC* in invasion and survival. The data showed that the numbers of wild‐type B11, AM73, and AC73 that invaded macrophages were 2.1 × 10^5^ CFU/ml, 2.3 × 10^5^ CFU/ml, and 2.0 × 10^5^ CFU/ml, respectively, at 0 hr and that 1.4 × 10^5^ CFU/ml, 1.4 × 10^4^ CFU/ml, and 1.0 × 10^5^ CFU/ml, respectively, survived for 1 hr in macrophages. The survival rates of the wild‐type B11 and AC73 were 64.5% and 48.6%, respectively, whereas the survival of AM73 was only 6.1% at 1 hr (Figure 8). The results at 0 hr and 1 hr indicated the invasion and intracellular survival ability of the strains. The results showed that the number of intracellular AM73 was slightly, but not significantly, larger than the number of B11 at 0 hr. However, the data at 1 hr suggested a significantly impaired survival of AM73 in host macrophages. These results showed that *acuC* plays a vital role in the survival of *A. hydrophila* in host macrophages.

### 3.10 Fluorescence microscopy analyses

**Figure 9:**
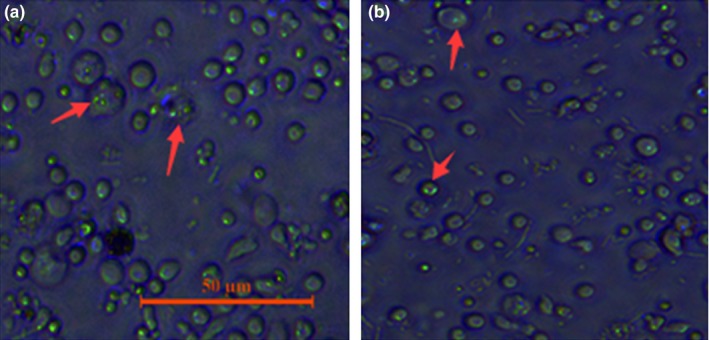
The status of wild‐type B11 labeled with EGFP in macrophages. Red arrows point to cells. (a) invasion; (b) intracellular survival after 1 hr

Fluorescence microscopy and labeled bacteria were employed to evaluate the status of *A. hydrophila* B11 in macrophages. The pictures taken at 0 hr (Figure 9a) and 1 hr (Figure 9b) show the invasion and intracellular survival, respectively. Numerous bacterial cells were found in the macrophages at 0 hr, and most of the bacterial cells remained alive at 1 hr post invasion. These results suggested that the bacteria could invade and survive in the macrophages.

### 3.11 Infection of *Danio rerio*

**Figure 10:**
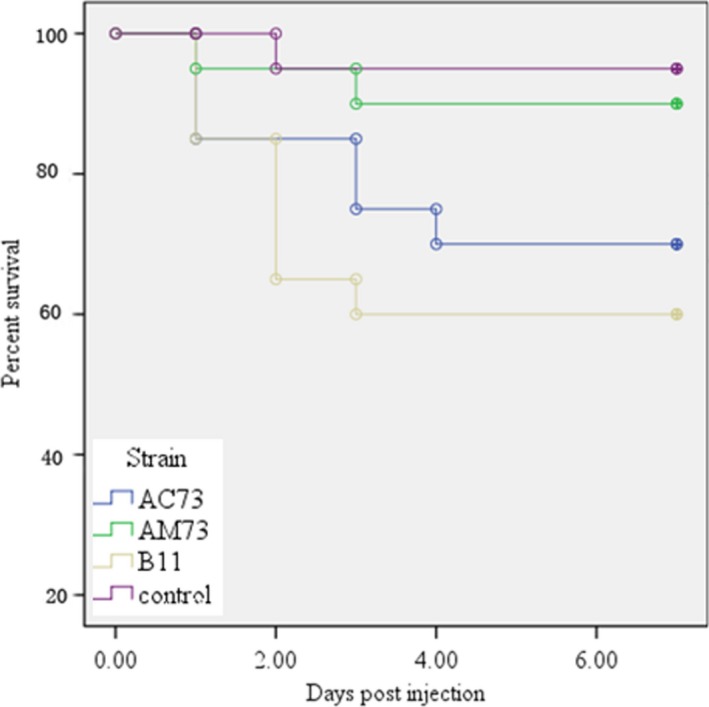
Infection of *Danio rerio* (*n* = 20) with wild‐type B11, the mutant AM73, and the complemented AC73 bacteria. Survival/mortality was monitored for 7 days. The *X*‐axis represents the days, and the *Y*‐axis represents the percent survival. The data were statistically evaluated and analyzed by the log‐rank test using GraphPad Prism 4 software (*p *<* *.05)

The defects in biofilm formation, adherence and intracellular survival of the mutant indicated that *acuC* plays an important role in the pathogenesis of *A. hydrophila*. To test this conclusion, we infected the *Danio rerio* with *A. hydrophila*. *Danio rerio* infected intraperitoneally with wild‐type B11 or AC73 showed 40% and 30% mortality in 3–4 days, whereas the virulence of the mutant AM73 was significantly lower (*p *=* *.019). After 7 days of observation, only 10% mortality was observed in the *Danio rerio* infected with AM73 (Figure 10). The bacteria isolated from the infected fish were identified by PCR as the wild‐type B11 and AM73. This result demonstrated that the mutation of *acuC* in AM73 significantly impaired the lethality of *A. hydrophila*.

### 3.12 Expression of various virulence genes

**Figure 11:**
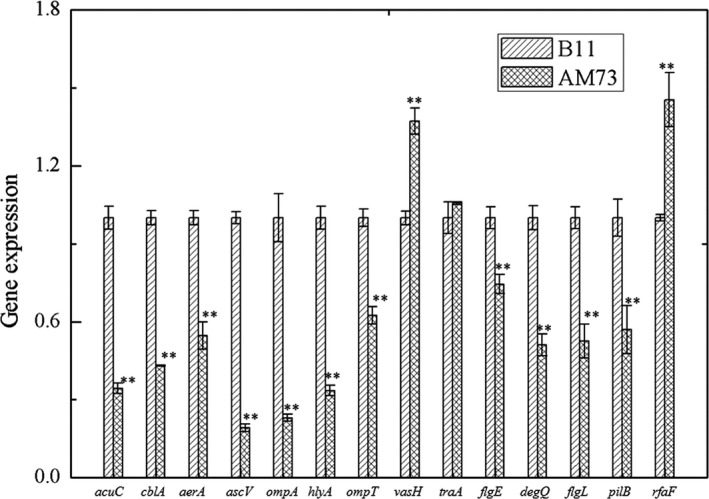
Expression profiles of *A. hydrophila* virulence‐related genes assayed by qRT‐PCR. The expression levels of *acuC* and 13 virulence genes in the wild‐type strain and AM73. The 16S rRNA was used as the internal control. Each bar represents the mean value of quintuplicates ± SD. Values denoted by different numbers of asterisks were significantly different when compared by ANOVA (“**” *p *<* *.01; “*” *p *<* *.05)

Quantitative real‐time reverse transcription‐PCR showed that a lipopolysaccharide gene (*rfaF*) and one of the type VI secretion system‐related protein genes (*vash*) were significantly up‐regulated in AM73, while the other type VI secretion system‐related protein gene (*traA*) showed no obvious change. The expression of two outer membrane protein genes (*ompA* and *ompTS*), the type Ш secretion system gene (*ascV*), pilin and flagellar family protein genes (*flgE*,* flgL*, and *pilB*), the serine peptidase gene (*degQ*), the hemolysin gene (*hlyA*), the adhesin subunit gene (*cblA*), and the aerolysin gene (*aerA*) were significantly down‐regulated in AM73 (Figure 11).

## 4 Discussion

In this study, a pathogenic strain of *A. hydrophila* B11 was found to invade the macrophages of *Tilapia mossambica* in vitro, and many of the bacteria survived in the macrophages for at least 24 hr. Once the bacteria entered the phagocytes, they must resist disruption by various enzymes present in the cells. The ability to survive in cells, even briefly, might open a window of opportunity for *A. hydrophila* and other pathogens to survive and trigger disease.

In this study, a mutant library of *A. hydrophila* was constructed by introducing the mini‐*Tn10* transposon into the bacteria. The mutant strain AM73 exhibited the poorest survival in macrophagocytes. The mutant gene was found to have the highest homology with a histone deacetylase/AcuC/AphA family protein gene of *A. hydrophila* subsp. *hydrophila* ATCC 7966. AcuC was found to be an intracellular protein by western blotting assay. The results of further assays showed that the enzymatic activity of the intracellular protein in AM73 was significantly reduced. These results demonstrated that the *acuC* gene, which encodes the histone deacetylase enzymatic activity, was mutated in AM73.

The protein acetylation/deacetylation posttranslational modification is an efficient mechanism for controlling the activity of structural proteins, gene expression regulators, and enzymes in response to rapidly changing physiological conditions (Gardner et al., 2006; Hu, Lima, & Wolfe, 2010). In *Bacillus subtilis*, the activity of acetyl‐coenzyme A (Ac‐CoA) synthetase is regulated by the protein acetylation/deacetylation posttranslational modification system comprised of AcuA and AcuC (Leipe & Landsman, 1997). OatA, a peptidoglycan O‐acetyltransferase, has been shown to be involved in *Listeria monocytogenes* immune escape and is critical for virulence (Aubry et al., 2011). In *Legionella pneumophila*, histone acetylation was demonstrated to be important for virulence (Schmeck et al., 2008). In this study, the deletion of *acuC* was found to reduce the intracellular survival ability of *A. hydrophila*. This result is consistent with the previous findings of Aubry et al. and Schmeck et al.

Quantitative real‐time reverse transcription‐PCR showed that deletion of *acuC* resulted in the up‐regulation of *vash* and *rfaF* and the down‐regulation of 10 genes, including *ompA, ompTS*,* traA*,* flgE*,* flgL*,* pilB, degQ, cblA, hlyA*, and *aerA*. These genes are closely related to the adhesion and virulence of *A. hydrophila* (Ho, Sohel, & Schoolnik, 1992; Qin et al., 2014; Quinn et al., 1994). Flagellar motility has been demonstrated to be necessary for *A. hydrophila* adhesion (Qin et al., 2016). Outer membrane proteins are important adhesion factors and protective antigens closely related to the virulence of *A. hydrophila* (Kawai, Liu, Ohnishi, & Oshima, 2004). OmpA has also been reported to be correlated with the adhesion of *A*. *hydrophila* (Quinn et al., 1994). *FlgE*, the structural gene encoding the flagellar hook protein, has also been reported to be correlated with the adhesion of *A. hydrophila* (Qin et al., 2014). It has previously been reported that the pilin gene, the gene coding for the flexible pilus subunit, was correlated with adhesion in *A. hydrophila* (Ho et al., 1992). In this study, the adhesion ability of *A. hydrophila* was significantly inhibited after deletion of *acuC*; this result is consistent with the gene expression results.

Biofilm formation has previously been reported to occur in three main stages: (1) attachment, specific protein‐based binding to abiotic surfaces; (2) proliferation and formation of mature biofilm structures; and (3) detachment, also called dispersal (O'Toole, Kaplan, & Kolter, 2000). Biofilm assays showed that the mutant strain AM73 was significantly defective in forming biofilms. This result indicated that acuC is involved in biofilm formation by *A. hydrophila*.

Unlike the well‐studied bacterial pathogenic processes, information on the intracellular survival of *A. hydrophila* is still very limited. Qin et al. (2014) found that a Δ*flgE* mutant of *A. hydrophila* showed significantly reduced intracellular survival in phagocytes. In this study, the intracellular survival of AM73 was significantly lower than the survival of the wild type. In addition, the expression of motility‐related genes, including *flgE*,* traA*,* flgL,* and *pilB* was significantly down‐regulated in AM73. Previous studies have shown that bacterial motility affects bacterial survival in phagocytes (Qin et al., 2014). In this study, the expression of *rfaF* was significantly down‐regulated in AM73. The *rfaF* gene is associated with the synthesis of lipopolysaccharide heptosyltransferase II. Guo et al. (1997) found that the proteins of lipopolysaccharide modification contribute to bacterial survival in macrophages by conferring resistance to antimicrobial peptides and nutrient scavenging. The expression of *ascV*, one of the type III secretion system genes, was also significantly down‐regulated in AM73. Some previous reports have revealed that the secreted molecules of the type III secretion system affect bacterial internalization (Cirillo, Valdivia, Monack, & Falkow, 1998).

In this study, the deletion of *acuC* resulted in the reduction in mortality of infected *Danio rerio*. The deletion of *acuC* also resulted in the down‐regulation of several genes, including *hlyA,* in the mutant strains. A hemolytic toxin, the product of *hlyA*, has also been reported to induce bacterial infection (Qian, Chen, Shen, & Shen, 1995). The results indicated that *acuC* is involved in the pathogenicity of *A. hydrophila* by affecting the expression of other virulence genes.

The significant reduction in the number of bacteria surviving in the host suggests a close relationship between *acuC* and survival, although the mechanisms of *A. hydrophila* survival in host phagocytes remain unclear. Further study is needed to reveal the form of *A. hydrophila* B11 residing in the macrophages and the activities of *A. hydrophila* B11 used to facilitate survival.

In conclusion, this study revealed that *A. hydrophila* B11 is able to invade the macrophages of *Tilapia mossambica* in vitro and that the bacteria can also survive more than 24 hr. The *acu*C gene can influence the expression of crucial virulence genes and biofilm formation, adhesion, and mortality in *A. hydrophila*, which indicates that AcuC is an important regulatory protein that contributes to the survival and pathogenicity of *A. hydrophila*.

## Conflict of Interest

None declared.
